# Molecular Defense Response of Pine Trees (*Pinus* spp.) to the Parasitic Nematode *Bursaphelenchus xylophilus*

**DOI:** 10.3390/cells11203208

**Published:** 2022-10-13

**Authors:** Inês Modesto, André Mendes, Isabel Carrasquinho, Célia M. Miguel

**Affiliations:** 1 Instituto de Tecnologia Química e Biológica António Xavier, Universidade Nova de Lisboa, Av. da República, 2780-157 Oeiras, Portugal; 2 Instituto de Biologia Experimental e Tecnológica, Avenida da República, Estação Agronómica, 2780-157 Oeiras, Portugal; 3 Department of Plant Biotechnology and Bioinformatics, Ghent University, Technologiepark 71, 9052 Ghent, Belgium; 4 BioISI—Biosystems & Integrative Sciences Institute, Faculdade de Ciências, Universidade de Lisboa, Campo Grande, 1749-016 Lisboa, Portugal; 5 Instituto Nacional de Investigação Agrária e Veterinária, Av. da República, Quinta do Marquês, 2780-157 Oeiras, Portugal; 6 LEAF—Linking Landscape, Environment, Agriculture and Food, Instituto Superior de Agronomia, Universidade de Lisboa, Tapada da Ajuda, 1349-017 Lisboa, Portugal

**Keywords:** pine wilt disease, differential gene expression, resistance, susceptibility, transcriptomics, migratory nematode, small RNAs, trans-kingdom RNA silencing, post-transcriptional regulation

## Abstract

Pine wilt disease (PWD) is a severe environmental problem in Eastern Asia and Western Europe, devastating large forest areas and causing significant economic losses. This disease is caused by the pine wood nematode (PWN), *Bursaphelenchus xylophilus*, a parasitic migratory nematode that infects the stem of conifer trees. Here we review what is currently known about the molecular defense response in pine trees after infection with PWN, focusing on common responses in different species. By giving particular emphasis to resistance mechanisms reported for selected varieties and families, we identified shared genes and pathways associated with resistance, including the activation of oxidative stress response, cell wall lignification, and biosynthesis of terpenoids and phenylpropanoids. The role of post-transcriptional regulation by small RNAs in pine response to PWN infection is also discussed, as well as the possible implementation of innovative RNA-interference technologies, with a focus on trans-kingdom small RNAs. Finally, the defense response induced by elicitors applied to pine plants before PWN infection to prompt resistance is reviewed. Perspectives about the impact of these findings and future research approaches are discussed.

## 1. Introduction

Pine wilt disease (PWD) is caused by the pine wood nematode (PWN) *Bursaphelenchus xylophilus*, a migratory endoparasitic nematode that infects the stem of conifer trees and affects mainly species of the genus *Pinus*. This nematode is transmitted to healthy trees through the insect vector *Monochamus* spp. during feeding on young branches. After entering the tree, PWN migrates through the resin canals and feeds on plant stem tissues, causing a progressive blockage of water flow and leading to tree death. Visible PWD symptoms include needle chlorosis and wilting [1,2].

In North America, where PWN is native, little damage is caused by PWD, as pine species are mostly resistant to the nematode infection [1,2]. However, in the regions where PWN was introduced, namely in Eastern Asia and Western Europe countries [2,3,4,5,6], several pine species have shown high susceptibility to PWD and it has been causing serious damage to forest ecosystems, as well as significant economic losses for the forest products industry [3].

To limit the spreading of PWD, several phytosanitary measures were implemented throughout the years. Presently, these consist mainly of detecting symptomatic trees, eliminating them, and burning or fumigating the wood [7]. Breeding for resistance can also be an effective long-term strategy to control the damage caused by pests and diseases [8,9]. The presence of genetic variability in survival to PWN inoculation in susceptible species indicates that it is possible to implement breeding programs for PWD resistance in these species [10,11,12]. In Japan and China, breeding programs have been successfully established for *Pinus thunbergii*, *Pinus densiflora,* and *Pinus massoniana* [10,13,14]. In Portugal and Spain, the first steps for the implementation of a similar breeding program for *Pinus pinaster* have also been taken [11,12,15]. The complexity of the breeding programs may be increased by the co-occurrence of several PWN lineages [16,17,18,19,20,21].

Different PWN pathotypes have been identified in Japan, China, and the Iberian Peninsula [16,17,18,19,20,21] and PWN isolates collected from the same geographic areas showed distinct virulence levels within the same species or between pine species [16,17,18]. Furthermore, genetic diversity of such isolates seems to be increasing in these regions over the years [22]. Therefore, the isolates used for inoculation assays should be carefully chosen, being previously tested for their virulence in the species of interest [16].

Although the molecular mechanisms involved in plant defense response to biotic stresses have been studied mainly in Arabidopsis and crop species, immune signaling pathways seem to be conserved across plant families [23,24]. The first level of plant defense is called pattern-triggered immunity (PTI) and initiates with the recognition by receptors localized on the cell surface (receptor-like kinases, RLKs, or receptor-like proteins, RLPs), of molecules known as pathogen-associated molecular patterns (PAMPs), conserved in large groups of pathogens, parasites, or pests, or of host-derived molecules resulting from plant cell damage, known as damage-associated molecular patterns (DAMPs) [23,25]. In the case of nematodes, defense is triggered by nematode-associated PAMPs (NAMPs), such as the pheromones ascarosides, chitin present in nematode eggshells and pharynx, and likely other molecules present on their surface coat [26]. The recognition of a foreign organism triggers a series of signaling events in the plant cell, including bursts of calcium and reactive oxygen species (ROS), as well as the activation of mitogen-activated and calcium-dependent protein kinases (MAPKs and CDPKs) [27]. Adapted pathogens can, however, suppress the plant’s immunity though the release of effectors. In turn, if these effectors are recognized by resistance genes, often intracellular nucleotide-binding/leucine-rich-repeat (NLR) receptors, the more robust defense response known as effector-triggered immunity (ETI) is initiated [28]. Although ETI is typically seen in response to biotrophic pathogens, relevant roles have been described for resistance genes, including NLR receptors, in achieving resistance to parasitic nematodes [29,30] and herbivorous insects [31,32]. Both the activation of PTI and ETI lead to a transcriptional reprogramming and the expression of defense response genes, including the activation of phytohormones pathways, such as salicylic acid (SA), jasmonic acid (JA), and ethylene (ET) [33].

In recent years, plant responses to PWN infection have been investigated at the molecular defense level, often resorting to differential gene expression analyses after inoculation in several pine species. In this review, we summarize the main findings from several molecular studies reported on different pine species focusing on common defense responses observed, including phytohormone signaling, secondary metabolism, oxidative stress, plant defense response, and resistance. For simplicity, we refer to resistance as including both tolerance and resistance responses [34]. Given the severity of this disease and its fast spreading across a vast geographical area, it is of great relevance to understand the basis of resistance to PWD. Therefore, we give a special emphasis to studies that compare the molecular responses of susceptible and resistant trees.

## 2. Defense Mechanisms Highlighted by the Transcriptional Response to PWN

Recently, several studies on gene expression after PWN inoculation have been published focusing on a variety of susceptible pine species and the resistant *P. pinea* and *P. yunnanensis* (Table 1). For *P. thunbergii* [35,36], *P. massoniana* [37], and *P. pinaster* [38] comparative analyses of differential gene expression were made between susceptible and selected resistant varieties or individuals. The experimental conditions varied among these studies, such as the age of the plants used (2 years old to adult trees), the use of seedlings or grafts, growth conditions (greenhouse, growth chamber, or field), the PWN isolates used (possibly with different levels of virulence), the amount of PWNs inoculated (500–30,000 PWNs), and sampling timepoints (Table 1). These differences may affect the plants’ response to PWN inoculation, as well as disease progression, and consequently gene expression, protein, and metabolite profiles. Nevertheless, comparing the transcriptional response to PWN inoculation in several pine species allowed for the identification of several common molecular mechanisms involved both in the susceptible and resistant responses.

### 2.1. Phytohormone Signaling

Phytohormones are signaling molecules with vital roles in plant development and response to stress, including response to biotic stresses. The recognition of a pathogen or pest and subsequent trigger of PTI or ETI by the plant cell leads to the accumulation of hormones, such as salicylic acid (SA), jasmonic acid (JA), and ethylene (ET) [50]. These three hormones are well described as having major roles in initiating downstream immune responses. SA is generally associated with response to biotrophic and hemi-biotrophic pathogens, while JA and ET have been associated with response to herbivory and necrotrophic pathogens, and the activation of these pathways is considered mutually exclusive [50,51]. However, this dichotomy is not always observed in species other than Arabidopsis, and JA has been implicated in the response to a wider range of pathogens and pests in monocots and gymnosperms [52]. Other hormones such as auxins, gibberellins, cytokinins, brassinosteroids or abscisic acid (ABA), which are usually linked to plant development or response to abiotic stresses, can also play roles in plant defense response against biotic stresses [53]. Despite the importance of phytohormones in plant immune response, little emphasis has been given to their role in pine response to PWN inoculation except in a few studies with *P. pinaster* [38,54]. 

The differential expression of genes encoding JA biosynthesis enzymes was observed in *P. pinaster* after inoculation, both in susceptible and resistant plants [38,46]. However, *12-oxophytodienoate reductase 3* (*OPR3*) was the only gene reported by both studies as being upregulated, soon after inoculation (24–72 h post-inoculation, hpi). Other *OPR*s, *allene oxide synthases* (*AOS*), *linoleate 13S-lipoxygenases* (*LOX*), and *phospholipase A2* (*PLA2G*), were upregulated in inoculated *P. pinaster* plants, particularly in resistant plants [38]. Genes responsive to JA were also induced after inoculation, such as the transcription factors *ethylene response factors* (*ERFs*), *jasmonate-ZIM domain* (*JAZ/Tify*) and *MYC2*, as well as the genes *pathogenesis-related protein 4* (*PR-4*), *pathogenesis-related protein 5* (*PR-5*), and *chitinases*, with higher levels of expression in resistant plants. This suggests that JA has an essential role in response to PWN in *P. pinaster* and may be important for resistance. Hormone quantification showed that JA levels were higher in inoculated plants when compared with controls at 72 hpi, but no differences were found in the more bioactive jasmonate-isoleucine (JA-Ile) [38]. On the other hand, Rodrigues et al. [54] reported higher levels of methyl jasmonate (MeJA) in susceptible plants at the same timepoint. Therefore, the higher activation of genes related to JA biosynthesis and response in resistant plants were not explained by higher levels of JA or the quantified conjugated forms. In other pine species, the upregulation of JA responsive genes after inoculation has also been reported. One *JAZ/Tify* transcription factor, the JA responsive *PR-3*, *PR-4*, and *PR-5*, and JA biosynthesis genes were upregulated in *P. massoniana* susceptible plants 24 hpi [14,41]. The genes *PR-2*, *PR-3*, *PR-4*, and *PR-5* were also upregulated in susceptible *P. densiflora* plants [44], as well as in susceptible and resistant *P. thunbergii* [35,36]. These results indicate that the JA pathway might have a central role in response to PWN in several pine species, but further studies are needed. Complex responses resulting from less-known crosstalk mechanisms between JA and other hormone signaling pathways may possibly help to explain its role in PWD.

In Arabidopsis, JA defense response consists of two antagonistic branches, regulated by distinct transcription factors, the MYC-branch and the ERF-branch [55,56]. While ET is necessary for the activation of the ERF-branch, usually in response to necrotrophic pathogens, ABA inhibits this defense response and leads to the activation of the MYC-branch in response to herbivory [50,55,56,57]. ABA responsive genes were reported as upregulated in susceptible *P. pinaster* 24 hpi [48] and susceptible *P. densiflora* after inoculation [45], as well as in the resistant species *Pinus yunnanensis* [49]. In *P. pinaster*, the upregulation of *PYL4*, which encodes a protein crucial for the ABA–JA crosstalk, and *PP2C*, involved in ABA signaling, was only observed in resistant plants [38]. Furthermore, several *NAC* transcription factor genes, involved in the ABA–JA crosstalk, were more expressed in resistant plants. This seems to suggest a role for ABA in response and resistance to PWN. On the other hand, *ERF* genes were also upregulated in *P. pinaster* at 72 hpi [38] and two *1-aminocyclopropane-1-carboxylic acid (ACC) synthases*, encoding an enzyme essential in the synthesis of ET, were upregulated in *P. yunnanensis* 24 hpi [49], suggesting a role for ET in PWN defense response. ET quantification has been reported for *P. thunbergii*, which showed an increase in ET levels only 2 weeks after inoculation. Increased ET levels were associated with the formation of embolisms in the xylem and consequent wilting symptoms [58,59]. In turn, ABA quantification in *P. pinaster* showed that, although no differences were observed between resistant, susceptible, and control plants at 72 hpi [38,54], ABA levels were lower in resistant plants at 48 hpi [54]. Therefore, the importance of ABA and ET pathways in pine response to PWN are still unclear. 

The upregulation of genes with roles in the synthesis and accumulation of SA, namely *enhanced disease susceptibility 1* (*EDS1*), *phytoalexin deficient 4* (*PAD4*) and *senescence associated gene 101* (*SAG101*), as well as several SA responsive genes, such as *WRKY* transcription factors (e.g., *WRKY23*, *WRKY50*, *WRKY51*) and *PR-1*, was observed in susceptible *P. pinaster* plants 72 hpi [38]. Furthermore, SA quantification revealed that susceptible *P. pinaster* plants had significantly higher levels of this hormone at 48 hpi and 72 hpi [38,54], suggesting that the SA pathway is associated with *P. pinaster* susceptibility to PWN. *WRKY* transcription factors, namely *WRKY6* and *WRKY51*, were reported also as upregulated in susceptible *P. densiflora* trees [45], while *PR-1* genes were more expressed in susceptible *P. thunbergii* plants when compared to resistant ones [35,36], suggesting that the activation of the SA pathway may also have a role in susceptibility in pine species other than *P. pinaster*. The activation of the SA defense pathway may inhibit JA response, in a manner that is independent of JA levels [51], as SA seems to suppress the JA defense response mostly at the transcription level in Arabidopsis. In this model species, SA influences the activity and localization of transcriptional regulators with roles in JA signaling and induces negative regulators of JA responsive genes, such as the WRKY transcription factors. Although further research is needed to understand whether SA inhibits the JA pathway in susceptible pine plants, SA/JA crosstalk may have an important role in the outcome of infected pine trees, with the activation of the SA defense pathway as a crucial step for susceptibility. 

Overall, the role of phytohormones in PWN response and resistance needs further clarification in the pine species of interest. Quantification of hormones at several timepoints after PWN inoculation, as well as investigating the effect of hormone application in the plant response, might help in understanding their relevance in PWD.

### 2.2. Secondary Metabolism

The importance of secondary metabolites in plant defense response, and in particular in conifers’ defense response, has been well described for the interaction with several pests and pathogens [60,61,62]. The expression of genes encoding secondary metabolites’ biosynthetic enzymes in response to PWN inoculation has also been reported for a number of pine species. Quantification of these metabolites in PWD-susceptible and -resistant species has been described, as well as the effect of some of these compounds on PWN mobility and survival (Table 2). Secondary metabolites include terpenes and phenylpropanoids, among other compounds.

#### 2.2.1. Biosynthesis of Terpene Compounds

The upregulation of genes involved in the terpene backbone biosynthesis pathway after PWN inoculation has been reported for *P. massoniana* [37], *P. pinaster* [38,46,48,49], *P. pinea* [48], and *P. yunnanensis* [49]. For instance, *hydroxymethylglutaryl-CoA reductase* (HMGCR) was upregulated both in *P. massoniana* [37] and *P. pinaster* [38,46], while *1-D-xylulose-5-phosphate synthase* (*DXS*) was upregulated in *P. massoniana* [37], *P. pinaster* [46,48,49], and *P. yunnanensis* [49], and *1-hydroxy-2-methyl-2-(E)-butenyl-4-diphosphate synthase* (*HDS*) was upregulated in *P. massoniana* [37] and *P. pinea* [48]. 

Several terpene synthases genes were more expressed in resistant *P. massoniana* plants than in susceptible ones after PWN inoculation, including *(-)-limonene synthase*, *(-)-*β*-pinene synthase*, *(+)-α-pinene synthase,* and *longifolene synthase* [37,63]. Two of these terpene synthases, namely α-pinene (*PmTPS4*) and longifolene (*PmTPS21*) synthases, were further characterized [63]. The enzyme α-pinene synthase produced the monoterpenes α-pinene, β-pinene, β-myrcene, and D-limonene, while longifolene synthase produced the sesquiterpene longifolene and the monoterpene α-pinene. All of these compounds had an inhibitory effect on PWN survival when applied separately to PWN in vitro cultures, and a stronger effect when applied in combination. Therefore, the higher expression levels of terpene synthase genes probably result in the increased synthesis of terpene compounds with nematicidal effect, leading to resistance in *P. massoniana*. Other terpene compounds extracted from *P. massoniana* were shown to have a repellent effect on PWN (Table 2) [64]. However, no information about the concentration of such terpene compounds in resistant or susceptible *P. massoniana* plants is yet available.

In other pine species, the upregulation of terpene synthases genes after PWN inoculation was also observed. Shin et al. [44] reported the upregulation of *limonene cyclase* in susceptible *P. densiflora* plants, while *α-pinene synthase* was upregulated in the resistant species *P. pinea* [65]. In *P. pinaster*, genes involved in the synthesis of diterpenes, such as *bifunctional abietadiene synthase* (*AS*) and *bifunctional levopimaradiene synthase* (*LPS*), as well as genes involved in the sesquiterpenes biosynthesis pathway, such as *(-)-germacrene D-synthase* (*GERD*), had higher expression levels in resistant plants than in susceptible ones [38].

**Table 2:** Secondary metabolites with a toxic effect on pinewood nematode (PWN).

Secondary Metabolite	Type of Compound	Species of Origin	Effect on PWN	References
α-humulene	Sesquiterpene	*Pma*	repellent	[64]
Calarene	Sesquiterpene	*Pma*	repellent	[64]
β-bisabolene	Sesquiterpene	*Pma*	repellent	[64]
Dihydroabietane	Diterpene	*Pma*	repellent	[64]
α-pinene	Monoterpene	*Pma*	repellent	[64]
		*Pma*	nematicide	[63]
β-pinene	Monoterpene	*Pma*	nematicide	[63]
β-myrcene	Monoterpene	*Pma*	nematicide	[63]
D-limonene	Monoterpene	*Pma*	nematicide	[63]
Longifolene	Sesquiterpene	*Pma*	nematicide	[63]
Pinosylvin monomethyl ether (PME)	Stilbene	*Pma*, *Pst*, *Ppa*	nematicide	[64]
		*Pst*	nematicide	[66]
Dihydropinosylvin monomethyl ether (DPME)	Stilbene	-	nematicide	[64]
		*Pst*	nematicide	[66]
		*Pst*	nematicide	[67]
Pinosylvin	Stilbene	-	nematicide	[64]
Methyl ferulate	Phenolic compound	*Pma*	nematicide	[64]
Ferulic acid	Phenolic acid	*-*	nematicide	[64]
(-)-nortrachelogenin	Lignan	*Pma*	nematicide	[64]
(+)-pinoresinol	Lignan	*Pma*	nematicide	[64]

*Pma*—*Pinus massoniana*; *Pst*—*Pinus strobus*; *Ppa*—*Pinus palustris*; PWN—pinewood nematode.

Despite these reports of differential gene expression in inoculated pine plants, connecting it to increased levels of terpene compounds has proven difficult. For susceptible *P. pinaster* and *P. sylvestris* plants, or for the resistant *P. pinea*, *P. halepensis,* or *P. radiata*, no alterations were found in the concentration of volatile and non-volatile terpenes after inoculation with PWN [68,69] at any of the studied timepoints (3 hpi to 2 months post-inoculation). However, an increase in diterpenes and sesquiterpenes was observed in *P. pinea* and *P. halepensis* after mechanical wounding, mimicking the insect vector feeding [68], while a significant increase in *P. pinea* limonene concentration was observed after feeding by *M. galloprovincialis* [70]. In *P. pinaster*, feeding by *M. galloprovincialis* caused an increase in several terpene compounds in susceptible plants, mainly in β-pinene, α-pinene, β-caryophyllene, and germacrene D, while a slight increase in β-myrcene and limonene was observed [70]. As PWNs enter the tree stem through wounds made during *M. galloprovincialis* feeding, the amount of terpene compounds produced and their relative proportions after wounding may impact the success of PWN infestation and consequently the resistance/susceptibility phenotypic outcome after infection. Studies to link gene expression and the synthesis of terpene compounds are still missing. Moreover, characterizing the enzymes with terpene synthase functions encoded by genes upregulated after wounding or PWN inoculation, similar to what was described for two *P. massoniana* enzymes [63], would elucidate their relevance and the role of their products in resistance to PWN.

#### 2.2.2. Phenylpropanoids Biosynthesis

Phenylpropanoids have long been recognized for their roles in plant response to abiotic and biotic stresses, being key elements in resistance to pests and pathogens [62,71,72]. The phenylpropanoid biosynthesis pathway branches out into several pathways, such as flavonoid and stilbenoid biosynthesis. Therefore, this class of secondary metabolites includes a vast variety of compounds, such as flavonoids, isoflavonoids, anthocyanidins, stilbenes, tannins, suberin, lignans, and lignin [71]. The synthesis of these compounds is frequently induced by pathogens or pests in a large variety of plants. In PWD, several phenylpropanoids have been quantified in PWN-susceptible and -resistant species [66,69,73,74] and the effect of some of these compounds on PWN survival has been studied (Table 2) [64,66,67]. Furthermore, a large number of genes involved in phenylpropanoids biosynthesis were upregulated after PWN inoculation in various pine species, revealing its importance in pine response to PWN (Table 3).

The constitutive levels of total phenolic content have been measured in several resistant and susceptible pine species in an attempt to associate these levels with the phenotypic outcome after PWN inoculation [69,73,74]. However, high levels of phenolics have been found both in resistant species, such as *P. pinea*, *P. halepensis,* and *P. radiata*, and in the susceptible species *P. pinaster*. Furthermore, results were not in accordance across studies, possibly due to the use of plants with different ages (2–3 y.o. in Nunes da Silva et al. [69]; 12 y.o. in Trindade et al. [74]) and the collection of data at different timepoints (24–72 hpi in Pimentel et al. [73] and Trindade et al. [74]; 2 months post-inoculation in Nunes da Silva et al. [69]). Therefore, measurement of total phenolics seems of little value to discriminate between PWN-resistant and -susceptible phenotypes. Instead, the synthesis of specific phenolic compounds may be linked to resistance [64,66,74].

The first genes in the phenylpropanoid biosynthesis pathway, such as *phenylalanine ammonia-lyase* (*PAL*), *4-coumarate-CoA ligase* (*4CL*), *caffeoyl-CoA O-methyltransferase* (*CCoAOMT*) or *caffeic acid O-methyltransferase* (*COMT*), were upregulated in several susceptible pine species after PWN inoculation, namely in *P. densiflora*, *P. massoniana* and *P. pinaster* (Table 3) [38,40,44]. *PAL* and *4CL* were also upregulated in the resistant species *P. strobus* [66] and more expressed in *P. pinaster*-resistant plants than in susceptible ones [38], suggesting that the activation of the phenylpropanoid biosynthesis pathway is a common response to PWN and may be important in resistance.

Several genes of the flavonoid biosynthesis pathway, such as *chalcone synthases* (*CHS*), were also induced by PWN inoculation in the susceptible species *P. densiflora*, *P. massoniana*, *P. thunbergii,* and *P. pinaster*, as well as in the resistant *P. pinea* [36,38,39,40,44,46,48]. In *P. pinaster* and *P. densiflora*, a higher expression of flavonoid biosynthesis pathway genes was observed in resistant varieties [38,75], suggesting that the synthesis of flavonoids may have a role in PWN resistance. Accordingly, high-constitutive and PWN-induced levels of the flavonoids taxifolin and rutin were detected in the resistant species *P. halepensis*, while the susceptible *P. pinaster* and *P. sylvestris* had lower levels of these compounds [74]. Furthermore, levels of total flavonoids decreased in susceptible *P. massoniana* plants after PWN inoculation [41]. In many plant–nematode interactions, the activation of the flavonoid biosynthesis pathway has been associated with resistance to nematodes [72]. Products of this pathway have been shown to be toxic to several nematode species. For instance, naringenin, the product of CHS, caused a reduction in burrowing nematodes’ (*Radopholus similis*) egg hatching, while kaempferol and quercetin repelled both root-knot nematodes (*Meloidogyne incognita*) and burrowing nematodes [76]. These compounds, and others with roles in plant resistance to parasitic nematodes (see [72]), may also affect PWN. Therefore, the levels of flavonoids should be further investigated in resistant pine species and varieties, as well as the toxicity and repellent effect of such compounds on PWNs. 

*Pinosylvin synthase* (*STS*), a stilbene biosynthesis gene, was upregulated in *P. densiflora* and *P. pinaster* inoculated plants [44,46], and in the resistant species *P. yunnanensis* and *P. strobus* [49,66]. In *P. strobus*, an increase in the pinosylvin derivates dihydropinosylvin monomethyl ether (DPME) and pinosylvin monomethyl ether (PME) was observed together with *STS* upregulation, while in the susceptible species *P. koraiensis* and *P. densiflora* PME was not detectable and DPME was present only in trace amounts in *P. koraiensis* [66]. These compounds were shown to be toxic to PWN in in vitro assays, affecting nematode mobility and survival [64,66]. Interestingly, PME was more toxic to adult PWNs, while DPME was more toxic to juveniles [66]. High-constitutive levels of another stilbene, resveratrol, were observed in *P. pinea* and *P. halepensis* [74]. Levels of this compound also increased after PWN inoculation in *P. halepensis*. Therefore, the synthesis of stilbenoid compounds seems to be relevant in achieving resistance in some pine species. 

Genes involved in the synthesis of lignans were upregulated in *P. massoniana* [40], while the synthesis of (+)-seoisolariciresinol was induced by PWN inoculation in *P. halepensis* [66]. Furthermore, the lignans (-)-nortrachelogenin and (+)-pinoresinol have been shown to be nematicidal for PWN [64]. However, the role of lignan compounds in PWN response has not been much explored, both in resistant and susceptible pine species or varieties. 

Genes encoding transcription factors likely to be involved in the regulation of the phenylpropanoid, flavonoid and anthocyanin pathways, such as *bHLH*, *MYB,* or *WRKY* [71], were upregulated after PWN inoculation in *P. densiflora*, *P. pinaster*, *P. pinea*, *P. yunnanensis,* and *P. strobus* [38,45,49,66], supporting the importance of these pathways in pine response to PWN. However, most of the phenylpropanoid compounds reported were associated with resistance in one or few pine species. It is possible that each species may depend on a different combination of phenylpropanoids to achieve resistance, which may be species-specific. On the other hand, considering the existing variety of such compounds in plants, the lack of overlap between species may be simply due to the lack of extensive data. Further research focusing on the quantification of the several classes of phenylpropanoids before and after PWN inoculation, guided by the transcriptomics studies available, might provide new insights into the conserved and unique resistance mechanisms in the different pine species and varieties. 

#### 2.2.3. Lignin Biosynthesis and Cell Wall Reinforcement

Lignin is another product of the phenylpropanoid biosynthesis pathway. Several genes encoding enzymes specific to lignin biosynthesis were upregulated in *P. densiflora*, *P. massoniana*, *P. thunbergii*, *P. pinaster,* and *P. yunnanensis* in the initial response after PWN inoculation (24–72 hpi) (Table 3) [35,37,38,44,46,49]. Moreover, higher expression of genes involved in this pathway seems to be associated with the resistance phenotype in susceptible pine species. For instance, *peroxidase* genes, encoding an enzyme involved in the last step of lignin synthesis, were more expressed in resistant plants of *P. massoniana*, *P. pinaster,* and *P. thunbergii* [35,37,38]. Higher levels of lignin in cell walls around the inoculation zone were in fact associated with resistance in *P. pinaster* and *P. thunbergii* [38,77]. Furthermore, this lignin accumulation has been linked to a limitation in PWN migration in *P. thunbergii* stem tissues [77]. Therefore, increased lignification around the inoculation zone seems to be a conserved defense mechanism in resistant varieties within susceptible pine species. This can interfere with PWN migration as observed in *P. thunbergii*, but possibly also with PWN ability to digest plant cell walls and feed on their content [26]. It remains to be elucidated if constitutive levels of lignin in the stem also vary within pine species and if these levels can also influence the plant’s phenotype after PWN inoculation. Cell wall reinforcement may also result from the cross-linking of the hydroxyproline-rich glycoproteins extensins (HRPG), catalyzed by peroxidases, which has also been associated with increased resistance to pathogens. Cross-linking of structural cell wall proteins such as extensins is the first histochemical modification observed in cell walls damaged by PWN, followed by lignification [77,78].

### 2.3. Oxidative Stress Response

During nematode infection, plants are typically under intense oxidative stress, where the reactive oxygen species (ROS) can be generated as part of the plant defense mechanisms, by dying plant cells, or by the nematodes themselves [26]. After nematode recognition, ROS produced by the plant act as signaling molecules in the activation of the defense response, having a role in the strengthening of plant cell walls via cross-linking, and may have a toxic effect on nematodes [25,26]. However, ROS are toxic to plant cells and may lead to their death if not transformed into innocuous molecules. Accordingly, during PWN infection, genes involved in ROS detoxification were differentially expressed after PWN inoculation in *P. densiflora*, *P. massoniana*, *P. thunbergii*, *P. pinaster,* and *P. yunnanensis* (Table 4) [35,36,37,38,40,44,46,49]. Furthermore, a few genes were reported to be more expressed in resistant than in susceptible plants, such as *peroxidases* [35,37,38], *catalases* [36,37], *glutathione S-transferase* [38], *superoxide dismutase,* and *glutathione reductase* (Table 4) [37]. In accordance with these gene expression results, the proteins glutathione S-transferase and superoxide dismutase were detected in higher levels in resistant *P. massoniana* plants 2 weeks post-inoculation [42].

Measurements of ROS in *P. massoniana* revealed that, after PWN inoculation, levels of superoxide anion (O_2_^−^) and hydrogen peroxide (H_2_O_2_) increased at an early stage of the infection (24 hpi) for both resistant and susceptible plants, and gradually decreased at 15 to 30 days post-inoculation (dpi) only in resistant plants [37]. At 24 hpi, the levels of H_2_O_2_ were higher in resistant plants when compared with susceptible ones, which was inverted at 15 and 30 dpi, when a steep increase was observed in susceptible plants. Thus, there seems to be a more efficient ROS detoxification in resistant plants, especially in more advanced phases of the disease, probably due to the action of the enzymes encoded by the genes more expressed in resistant *P. massoniana* plants.

Although ROS quantification has not been reported for other pine species infected with PWN, it is plausible that similar processes have a role in their resistance. In *P. pinaster*, genes encoding for aldehyde oxidase (GLOX) enzymes, which produce H_2_O_2_, had considerably higher expression levels in resistant plants in an early stage of infection (72 hpi) [38], which is consistent with the higher levels reported for resistant *P. massoniana* in a similar stage of infection (24 hpi). The production of H_2_O_2_ in this early stage may promote the cross-linking in the plant cell wall, increasing its strength and impairing PWN migration and feeding.

At the metabolomics level also, the importance of cell redox homeostasis in resistance has been highlighted [47]. *Pinus pinaster*-resistant plants accumulated osmolytes after inoculation (e.g., fucose, GABA, trehalose), which are involved in protecting the cells from oxidative damage. Considering the importance that ROS detoxification seems to have in achieving resistance, it would be interesting to measure ROS at several timepoints after PWN inoculation in several pine species of interest, comparing resistant and susceptible varieties. Quantifying the enzymes encoded by the differentially expressed genes reported and correlating them with ROS concentration should highlight which enzymes are important for pine response and resistance to PWN.

### 2.4. Plant Defense Response Genes

Pathogenesis-related proteins are induced by pathogens or pests as part of the host plant defense. They comprise a variety of proteins with different properties and functions, such as chitinases (PR-3, PR-4, PR-8, and PR-11), thaumatin-like proteins (PR-5), and proteinase inhibitors (PR-6). Although some of these proteins have been associated with resistance to specific pathogens, PR proteins are thought to be part of a generalized plant defense response to a broad range of pathogens and pests, even though not always effective [79]. During PWN infection, the expression of several *PR* genes is induced in pines trees, including *PR-1* (unknown function; *P. thunbergii*, *P. massoniana*, *P. pinaster*), *PR-2* (beta-1,3-glucanase-like proteins; *P. thunbergii*, *P. densiflora*), *PR-3* (chitinases; *P. thunbergii*, *P. densiflora*, *P. massoniana*), *PR-4* (chitinases or chitin-binding proteins; *P. thunbergii*, *P. densiflora*, *P. massoniana*, *P. pinaster*), *PR-5* (thaumatin-like proteins; *P. thunbergii*, *P. densiflora*, *P. massoniana*, *P. pinaster*, *P. pinea*), *PR-6* (proteinase inhibitors; *P. thunbergii*), *PR-10* (ribonuclease-like proteins; *P. thunbergii*, *P. densiflora*, *P. massoniana*), and *PR-14* (lipid-transfer proteins; *P. massoniana*, *P. pinaster*, *P. pinea*) [35,36,37,38,40,44,48]. Although the role of these proteins in PWN resistance in unknown, some of these genes were more expressed in resistant pine varieties.

The chitinases *PR-3* and chitin-binding *PR-4* were more expressed in resistant *P. thunbergii* [36] and *P. pinaster* [38] when compared to susceptible plants. Chitin is a main component of nematode eggshell [26,80] and possibly the pharyngeal lumen walls [29], suggesting that chitinases may compromise egg integrity and embryo development, as well as PWN feeding. Treatment with chitinase plant extracts caused premature egg hatching and increased juvenile mortality in the root-knot nematode *Meloidogyne hapla* [81]. Assessing the effects of chitinase extracts from pine trees, especially extracts from resistant varieties, in the several life stages of PWN would elucidate their role in PWD resistance. *PR-5*, which has antifungal activity, and *PR-10* were also more expressed in resistant *P. pinaster* [38] and *P. thunbergii* [35] plants, respectively. It is unknown if these proteins have an impact on nematode growth, multiplication, or spread. Evaluating the effects of pine extracts of these proteins, similarly to what is here suggested for chitinases, would clarify this topic.

Other genes previously associated with plant defense response, such as *mannose/glucose-specific lectin* and *ricin B-related lectin*, were also upregulated early after inoculation (24–72 hpi in *P. pinaster*, *P. pinea,* and *P. massoniana*) [40,48]. Plant lectins have been shown to interact with mono- or oligosaccharides from several pests and pathogens and some were reported as toxic [82]. Interestingly, some lectins are involved in *A. thaliana* defense response against the root-knot nematode *M. incognita* [83]. 

### 2.5. Resistance Genes

Plant resistance genes are receptors that detect effectors released by a pathogen, parasite, or pest, starting a highly specific defense response that is effective in stopping the spread and multiplication of the invading organism. Resistance genes include the membrane receptors RLKs or RLPs, which recognize apoplastic effectors, and more often the intracellular receptors NLRs, which identify cytoplasmic effectors. These receptors may interact with the effector directly or indirectly by monitoring alterations caused by the effector in a plant co-factor [28,30]. Several studies have shown important roles of resistance genes in plant interactions with parasitic nematodes and herbivorous insects [29,30,31,32]. For instance, the RLP receptor Cf-2 recognizes the potato cyst nematode *Globodera rostochiensis* apoplastic effector venom allergen-like protein 1 (VAP1), indirectly, while the NLR receptor Gpa2 recognizes the potato cyst nematode *Globodera pallida* cytoplasmic effector GpRBP-1 [30]. The *VAP1* gene was also identified in the PWN genome and the knockdown of this gene resulted in significantly lower PWN migration in the stem of pine seedlings when compared to wildtype PWN [84]. This suggests that VAP1 also acts as an effector in PWN-pine interactions by suppressing pine defense response. Another apoplastic effector, BxSapB1, has also been described recently [85], without which PWN has lower virulence than wildtype PWN. Furthermore, a NAMP has been characterized, namely BxCDP1, which leads to the initiation of PTI in a brassinosteroid-insensitive 1-associated kinase 1 (BAK1)-dependent manner [86].

The receptors involved in the recognition of these NAMP and effectors, and subsequent activation of pine defense response, are unknown. It is possible that different receptors participate in the activation of the immune response in resistant and susceptible plants, or that resistance genes detect PWN effectors and activate the more robust ETI in resistant plants. This is supported by the differential expression of distinct *RLK/RLP* and *NLR* receptor genes after PWN inoculation when comparing resistant and susceptible *P. pinaster* plants, as well as the downregulation of *NLRs* and resistance genes in susceptible *P. massoniana* [38,41]. Furthermore, two NLR receptor proteins were constitutively more expressed in resistant *P. massoniana* plants [43]. The activation of the ETI usually takes place in plants adapted to the pathogen or pest, implying a coevolution of the two organisms and often an arms race [28]. As PWN is an invasive parasite, susceptible pine species have not evolved in the presence of PWN. Therefore, genetic resistance to PWD occurring in natural stands has likely evolved due to the selective pressures of another pathogen or pest. In fact, one gene may confer resistance to more than one organism, as in the case of *Mi-1.2*, which confers resistance to root-knot nematodes (*Meloidogyne* spp.) [87], the potato aphid (*Macrosiphum euphorbiae*) [88], the white fly (*Bemisia tabaci*) [89], and the tomato psyllid (*Bactericera cockerelli*) [90]. To better understand pine response and resistance to PWN, it would be interesting to investigate if ETI is activated in resistant plants, what effectors may be recognized by resistance genes, and what receptors are involved in these responses.

## 3. Post-Transcriptional Regulation Mediated by Small RNAs in Pine Response to PWN

Small RNAs (sRNAs) are a class of non-coding RNAs, with 20 to 35 nucleotides, that are key players in post-transcriptional and transcriptional gene silencing [91]. MicroRNAs (miRNAs), one of the best studied sRNA classes, are mostly involved in post-transcriptional gene silencing by guiding the cleavage or translation inhibition of complementary target transcripts [92] with roles in a variety of processes, including plant development, as well as response to abiotic and biotic stresses [93,94]. During plant defense response, sRNAs are known to be involved in the regulation of plant hormone synthesis and signaling, callose deposition, expression of NLR receptors, and of other resistance proteins, ROS detoxification, and secondary metabolites synthesis [95], being essential players in PTI and ETI immune responses. After nematode infection, several host sRNAs have also been associated with resistance traits. Transcription factors and hormone signaling genes are examples of sRNA targets associated with plant defense mechanisms against parasitic nematodes [96,97]. The great majority of studies on sRNAs involved in plant response to nematode diseases focus on sedentary endoparasitic nematodes and their involvement in feeding sites’ development [96,98,99,100]. Nevertheless, the first steps have been taken to understand the role of miRNAs in PWD response.

### 3.1. MicroRNA-Mediated Response to PWN

The first report of miRNAs differentially expressed in response to PWD was in *P. massoniana* [101]. These authors showed that in *P. massoniana* inoculated with PWN, several miRNAs were differentially expressed in the needles during the first 3 days post-inoculation (dpi). The predicted targets for these miRNAs were associated with plant hormone signaling (e.g., zeatin synthesis and ethylene signaling), RNA transport, splicing, and fatty acid metabolism, among other processes. The expression of hormone signaling predicted targets showed a significant increase at 2 dpi followed by a decrease at 3 dpi and negatively correlated with the expression of their corresponding miRNAs. The impact of the infection on hormone synthesis and accumulation was confirmed by the quantification of indole acetic acid (IAA) and zeatin contents in *P. massoniana* needles, which were shown to initially decrease (3 dpi), then increase (9 dpi), and finally decrease significantly at 14 dpi [101]. The suppression of growth-related hormone signaling and synthesis at 14 dpi was suggested by the authors to be a consequence of the damage caused by PWN. On the other hand, the initial decrease in hormone content (3 dpi) likely results from growth-defense trade-offs, suggesting that the tree has relocated its energy for the defense response to the detriment of growth and development [102]. However, the expression of miRNAs involved in the regulation of plant immune response was not detected in this study, probably because only the pine needles were sampled, while PWN only infects stem tissues. 

A recent study focusing on the quantification of *P. pinaster* stem miRNAs has, in fact, identified different miRNAs which are predictably defense-related [103]. MicroRNAs differentially expressed after inoculation targeted JA responsive genes, *RLK* and *NLR* receptor genes, transcription factors *WRKY*, genes involved in ROS detoxification, such as *thioredoxins* and *peroxiredoxins*, and genes involved in terpene biosynthesis, such as *LPS*. When comparing resistant and susceptible plants, only 8 miRNAs were differentially expressed, and their targets included genes possibly involved in the activation of plant defense response, such as *RLKs* and *GDP-L-fucose synthase 2*, ROS detoxification and JA signaling pathway. Many of the post-transcriptionally regulated pathways and genes have been shown to be PWN-responsive in several pine species, as described above. Targeting of JA regulators by miR166, miR947, miRnovel_43f, and miRnovel_110; regulation of RLKs, essential for PTI activation, by miR166h, miR951f, and miR947f; increase of oxidoreductase activity in resistant plants possibly involving miR3627m; and increased terpene synthesis as a consequence of miRNAs’ differential expression, have been suggested as important post-transcriptional events for *P. pinaster* resistance to PWN. In addition, trans-acting short-interfering RNAs (tasiRNAs), a class of siRNAs derived from specific miRNA cleaved transcripts, have also been predicted to originate from miR11532 and miR947f activity [103]. The predicted targets for these tasiRNAs seem to be involved in plant hormone signal transduction, plant-pathogen interaction, and flavonoid biosynthesis pathways, adding another regulatory layer to the control of the defense response pathways.

### 3.2. Trans-Kingdom RNA Silencing in PWD

In recent years, great interest has been shown in trans-kingdom sRNAs and their potential for new plant protection technologies against pathogens and pests. These sRNAs can perform regulatory functions after being transferred between distantly related organisms [104]. Plant-originated sRNAs can target pathogen housekeeping genes, effectors, pathogenicity, or development-related genes, for instance, and the pathogen sRNAs can suppress host immunity [99,105]. Naturally occurring trans-kingdom sRNAs can, therefore, be considered an addition to the well-known innate immunity *zig-zag* arms race between hosts and pathogens [106].

Possible targets for PWN and *P. pinaster* miRNAs in *P. pinaster* and PWN transcriptomes, respectively, have been identified [103]. *Pinus pinaster* genes putatively regulated by PWN trans-kingdom miRNAs are involved in transcriptional processes, protein synthesis and assembly, and immune response-related processes (e.g., isoprenoid biosynthesis and regulation of ABA-activated signaling pathway). The targeting of those genes has been supported by degradome data and suggests that PWN enhances its pathogenicity by weakening *P. pinaster* cell functions and preventing the establishment of a timely and effective defense response. On the other hand, pine miRNAs may target PWN response to stimuli, transcriptional response, detoxification of plant xenobiotics (e.g., *cytochrome P450* or *epoxide hydrolase* genes) and digestion of plant tissues (e.g., *peptidases* and *lysosomal enzymes*) [103], possibly affecting nematode pathogenicity.

Trans-kingdom RNA silencing opens new doors for the employment of technologies that use RNA-interference (RNAi) to regulate plant resistance, such as host-induced gene silencing (HIGS), or spray-induced gene silencing (SIGS) [107]. In HIGS, pathogen genes are silenced by expressing complementary double-stranded RNAs (dsRNA) or artificial miRNAs in the host plant, while in SIGS, the dsRNA is sprayed on the infected plant, having similar silencing results. Interestingly, the silencing of some PWN genes predicted to be targeted by *P. pinaster* miRNAs through the external application of double-stranded RNA (dsRNA) efficiently reduced PWN pathogenicity (e.g., *cytochrome P450* [108,109,110]; the peptidases *cathepsin* [111] and *cysteine peptidase* BxCAT2 [112]). This may be particularly important in the control of more virulent strains of PWN. By comparing 4 different isolates of PWN, Shynia et al. [112] have identified Bx-GH30 and Bx-CAT2 as proteins highly secreted by virulent PWN isolates, showing their contribution to isolate virulence in a host species and possible association with pine wilt disease. These effectors are good examples of potential candidates to be targeted aiming at nematode control strategies, and other candidates have been reported (e.g., [112,113,114,115]). Although naturally occurring trans-kingdom RNAi transference was never observed for plant–nematode interactions, the transference of artificial RNAi from the plant host to root-knot nematodes, and subsequent gene silencing, has been previously reported (e.g., [116,117]). For instance, HIGS has been successfully used to knock down several effector genes in *M. incognita* [116,118]. Therefore, *P. pinaster* miRNAs that putatively target PWN pathogenicity genes should be further validated and their potential application in HIGS or SIGS should be investigated [104,107].

## 4. Defense Response Induced by the Application of Phytohormones and Secondary Metabolites

In recent years, the application of elicitors to pine trees in order to induce plant immunity prior to PWN infection has been investigated as a PWD control method [119,120,121,122]. Elicitors can be a variety of substances, such as plant hormones, purified molecules derived from pathogens or pests, or synthetic molecules, which induce plant defense response [123]. These substances have the potential to be used as biocontrol agents, as they are more economical and eco-friendly approaches than the traditional use of insecticides against the insect vector or trunk injection of nematicides. A few elicitors have been evaluated for PWD with positive results in reducing disease progression or PWN multiplication, namely methyl salicylate (MeSA) and chitosan [119,120,121,122]. 

The application of MeSA in *P. densiflora* and *P. thunbergii* seedling leaves in the form of spray one and two weeks before PWN inoculation significantly decreased disease progression when compared to seedlings without treatment [119,120,124]. Treatment of *P. densiflora* plants with MeSA and subsequent inoculation with PWN seem to induce genes and pathways similar to those previously associated with PWN resistance, such as *PR-1*, *PR-2*, *PR-5*, peroxidases, extensins, flavonoid biosynthesis genes, and genes involved in ROS detoxification [119,124]. The expression levels of these genes were much higher in plants treated with MeSA than in non-treated plants, reinforcing their importance in achieving resistance to PWD.

Similarly, the application of chitosan, a compound derived from chitin, in the soil increased *P. pinaster* resistance to PWN, as shown by the significantly lower number of PWNs observed in treated plants at several timepoints after inoculation when compared to untreated ones [121,122]. Chitosan application induced catalase activity, an enzyme involved in ROS detoxification, as well as the production of phenolic compounds, anthocyanins (flavonoids), carotenoids (terpenes), and lignin [122]. Therefore, application of chitosan and MeSA induced similar pathways that seem to be crucial for PWD resistance in pine trees.

On the other hand, the trunk injection of MeJA had a small effect on improving *P. densiflora* resistance to PWN [124]. However, spraying *P. massoniana* seedlings with MeJA seems to induce the production of diterpenes and deter the insect vector *Monochamus alternatus* from feeding on the stems of elicited plants [125]. These observations show that MeJA has also the potential to be used as a control compound at the level of the host–insect vector interaction. Further studies are needed to confirm that MeJA application has the same effect on other pine trees and insects of the genus *Monochamus.*

## 5. Conclusions and Future Perspectives

**Figure 1:**
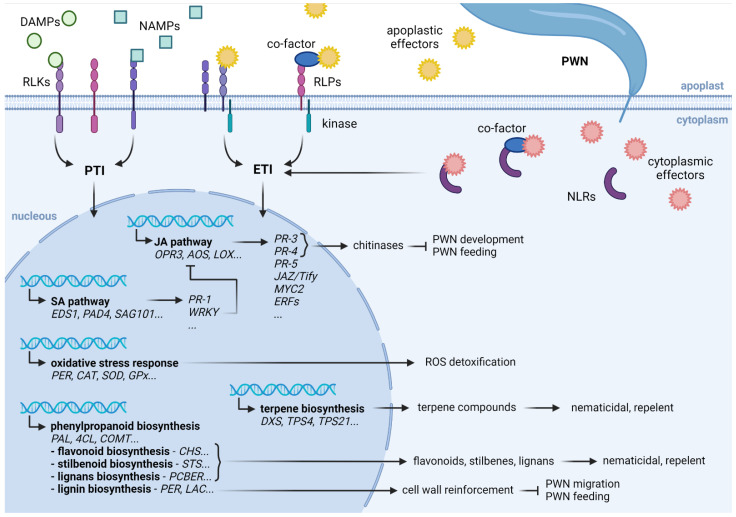
**Representation of pine defense response to pinewood nematode (PWN).** Pattern-triggered immunity (PTI) is initiated after recognition of nematode-associated molecular patterns (NAMPs), such as BxCDP1, or damage-associated molecular patterns (DAMPs) from damaged plant cells, by cell surface receptor-like kinases (RLKs) or receptor-like proteins (RLPs). PTI leads to a transcriptional reprograming of the plant cell, which includes the activation of hormone pathways, such as jasmonic acid (JA) and salicylic acid (SA) pathways, the expression of pathogenesis-related genes (PR), genes involved in oxidative stress response, and genes encoding enzymes involved in the biosynthesis of secondary metabolites (terpenes and phenylpropanoids). In susceptible plants, it is possible that the activation of the SA pathway inhibits the JA pathway. PWN releases effectors, such as the apoplastic VAP1 and BxSapB1, that repress the plant defense response. These effectors may be recognized by resistance genes, whether directly or indirectly through the monitoring of a co-factor protein that is altered by the effector. Effectors can be recognized by cell surface receptors, when they are apoplastic, or internal nucleotide-binding/leucine-rich-repeat (NLR) receptors, when they are injected into the cytoplasm by the PWN. Recognition of effectors by resistance genes initiates a stronger and more sustained defense response, the effector-triggered immunity (ETI).

After PWN infection, pine trees undergo a significant transcriptional change that may be followed by changes in the synthesis of proteins and metabolites (e.g. [47,63,66]). We presented an overview of the defense response pathways induced in several pine species by PWN inoculation, revealing that at least part of this defense seems to be conserved (Figure 1). More importantly, the analysis of more resistant pine varieties and families highlighted several resistance mechanisms, many of which were also conserved among pine species. ROS detoxification, limiting PWN migration and feeding through cell-wall reinforcement, including lignification, and production of secondary metabolites that affect PWN mobility, impairing PWN development and feeding through the action of chitinases, or directly influencing PWN survival by producing secondary metabolites with nematicidal effects, are likely to act together in achieving resistance to PWN. Small RNAs seem to be important players in regulating several of these pathways, as well as plant growth, during PWN infection. Moreover, naturally occurring trans-kingdom miRNA transference between PWN and its host may be an important process in repressing pine defense response or PWN pathogenicity. 

To better understand resistance mechanisms, it is important to complement transcriptomics studies with proteomics and metabolomics, since differences in mRNA levels do not always translate into protein abundance or activity due to variations in translational efficiency, which may be caused by post-transcriptional regulation, mRNA secondary structures or availability of cellular resources, and post-translational modifications, which influence protein structure or function. Extending these studies to a higher number of pine varieties and families selected for PWD resistance would allow us to distinguish between candidate genes specific to varieties from more general candidate genes. Functional analysis of candidate genes in model systems can confirm their role in resistance and give details about their functions [9]. After confirming their function, the overexpression, knockdown or knockout of the candidate genes may be explored aiming at obtaining resistance to PWN. However, genetically engineering conifer species is difficult, and many times optimized for a specific clonal background [126]. On the other hand, in many countries strict regulations prevent the plantation of genetically engineered trees in the fields. An alternative could be to use CRISPR/Cas9-directed genome editing technologies that are more leniently regulated in several countries, including Japan. Furthermore, genome editing may be more efficient and promising results have been obtained in *P. radiata* [127].

Furthermore, the search for SNPs in candidate genes for resistance and association with phenotype after PWN inoculation will be key to the development of molecular markers useful for accelerating breeding programs through marker-assisted selection. Developing biomarkers for resistance may also aid in the selection of trees with constitutive higher concentrations of specific secondary metabolites associated with resistance. The use of high-throughput phenomics could increase the precision, efficiency, and speed of the plant screening for resistant and susceptible phenotypes [128]. In combination with genomics and metabolomics, it would improve the identification of molecular markers and biomarkers. Interesting results have been obtained in detecting pine trees with PWD in the forest, using unmanned aerial vehicles with multispectral cameras, and machine learning algorithms [129]. The adaption of these methods in the screening of plant traits in large-scale inoculation assays could be of great relevance and should be further explored. In conclusion, the emerging knowledge on PWD is of great relevance for its control and should be explored in the near future taking advantage of the powerful high-throughput technologies and tools now available for precision plant breeding.

## Figures and Tables

**Table 1:** Details of the molecular response studies on pine trees infected with pinewood nematode (PWN).

Host Species	Type of Study	S/R	Type of Analysis	Time Points	Plant Material	Isolate and Origin	Inoc. PWNs	Expm. Conditions	Ref.
*P. thunbergii*	Gene exp. (LongSAGE)	S species, R varieties	S vs. R	72 hpi	2–3 yo seedlings	Shimabara, *P. thunbergii*	5000	Field	[10]
*P. thunbergii*	Gene exp. (SSH)	S species, R varieties	S vs. R	24 hpi, 72 hpi, 7 dpi, 14 dpi	2 yo grafts	Ka-4, *P. thunbergii*	10,000	Field	[35]
*P. thunbergii*, *P. massoniana*	Gene exp. (RNA-seq.)	S species	S response	24 hpi, 48 hpi, 72 hpi, 96 hpi, 5 dpi, 6 dpi	3 yo seedlings	Unknown	10,000	Greenhouse	[39]
*P. massoniana*	Gene exp. (SSH)	S species	S response	24 hpi, 72 hpi	3 yo seedlings	BXY03, *P. massoniana*	1500	Growth chamber	[40]
*P. massoniana*	Gene exp. (RNA-seq.)	S species, R varieties	S vs. R	24 hpi, 15 dpi, 30 dpi	4 yo ramets	Guangzhu-3B, unknown	10,000	Nursery	[37]
*P. massoniana*	Gene exp. (RNA-seq.)	S species	S response	24 hpi, 48 hpi, 72 hpi	2 yo seedlings	Unknown, *P. massoniana*	2000	Greenhouse	[41]
*P. massoniana*	Proteomics	S species	S vs. R	14 dpi	2 yo seedlings	Unknown	1000	Unknown	[42]
*P. massoniana*	Proteomics	S species	S vs. R	Constitutive response	grafts	-	-	Field	[43]
*P. densiflora*	Gene exp. (ACP, SSH)	S species	S response	21 hpi, 24 hpi, 7 dpi	4 yo and 8 yo seedlings	Unknown, *P. thunbergii*	6000 to 30,000	Field and nursery	[44]
*P. densiflora*	Gene exp. (RNA-seq.)	S species	Pathogenic vs. non-pathogenic PWN	28 dpi	adult trees (11–13 m)	Unknown, *P. densiflora*	60,000	Field	[45]
*P. pinaster*	Gene exp. (RNA-seq.)	S species	S response	6 hpi, 24 hpi, 48 hpi, 7 dpi	3 yo seedlings	Unknown	2000	Field	[46]
*P. pinaster*	Gene exp. (RNA-seq.)	S species, R varieties	S vs. R	72 hpi	4 yo seedlings	Bx013.003, *P. pinaster*	500	Greenhouse	[38]
*P. pinaster*	Metabolomics	S species, R varieties	S vs. R	14 dpi, 21 dpi, 28 dpi, 35 dpi	2 yo seedlings	Bx013.003, *P. pinaster*	500	Greenhouse	[47]
*P. pinaster*, *P. pinea*	Gene exp. (pyroseq.)	S and R species	S vs. R	24 hpi	2 yo seedlings	HF, *P. pinaster*	1000	Growth chamber	[48]
*P. pinaster*, *P. yunnanensis*	Gene exp. (RNA-seq.)	S and R species	S vs. R	6 hpi, 24 hpi, 48 hpi, 7 dpi	3 yo seedlings	Unknown	2000	Field	[49]

Exp.—expression; S—susceptible; R—resistant; hpi—hours post-inoculation; dpi—days post-inoculation; yo—years old; Inoc.—inoculated; Expm.—experimental; Ref.—references.

**Table 3:** Expression of genes related to secondary metabolism pathways in several pine species after inoculation with pinewood nematode.

Pathway	Genes	*P. densiflora*	*P. massoniana*	*P. thunbergii*	*P. pinaster*	*P. pinea*	*P. yunnanensis*	*P. strobus*
Phenylpropanoid biosynthesis	*phenylalanine ammonia-lyase (PAL)*	up			up; S < R			up
	*4-coumarate-CoA ligase (4CL)*	up			up; S < R			up
	*caffeoyl-CoA O-methyltransferase (CCoAOMT)*	up	up		up			
	*caffeic acid O-methyltransferase (COMT)*		up		up			
Flavonoid biosynthesis	*chalcone synthase (CHS)*	up	up	up	up; S < R	up		
	*chalcone isomerase*	up			up			
	*flavonol synthase (FLS)*				up			
	*flavonoid hydroxilase*	up						
	*leucoanthocyanidin dioxygenase (LDOX)*	up		up; S > R	up; S < R			
	*leucoanthocyanidin reductase (LAR)*				up; S < R			
	*anthocyanidin synthase (ANS)*				up; S < R			
Stilbenoid biosynthesis	*pinosylvin synthase (STS)*	up			up		up	up
	*pinosylvin O-methyltransferase (PMT)*							up
Lignans biosynthesis	*phenylcoumaran benzylic ether reductase*		up					
Lignin biosynthesis	*cinnamoyl-CoA reductase (CCR)*	up			up		up	
	*cinnamyl-alcohool dehydrogenase (CAD)*		up; down in S		up			
	*peroxidase (PER)*	up	up; S < R	up; S < R	up; S < R		up	
	*laccase (LAC)*				up; S < R			
Transcription factors	*bHLH*	up			up			up
	*MYB*	up			up		up	up
	*WRKY*	up			up	up		up
**References**		[44,45]	[37,39,40,41]	[35,36,39]	[38,46]	[48]	[49]	[66]

up—upregulated; S—susceptible; R—resistant.

**Table 4:** Expression of oxidative stress response genes that encode enzymes involved in detoxification of reactive oxygen species (ROS) after pinewood nematode inoculation in several pine species.

Genes	*P. densiflora*	*P. massoniana*	*P. thunbergii*	*P. pinaster*	*P. yunnanensis*
*superoxide dismutase*	up	down, S < R		up	up
*glutathione reductase*		down, S < R			
*glutathione peroxidase (GPx)*		up		up	
*L-ascorbate peroxidase*	up	down			
*catalase (CAT)*		down, S < R	S < R		
*catalase isozyme*		up			
*peroxidase (PER)*	up	S < R	S < R	S < R	up
*glutathione S-transferase*	up			up; S < R	up
*peroxiredoxin*	up				
*thioredoxin*	up			up	
**References**	[44]	[37,40]	[35,36]	[38,46]	[49]

up—upregulated; S—susceptible; R—resistant.

## Data Availability

Not applicable.
